# Pre-cachectic changes in amino acid homeostasis precede activation of eIF2α signaling in the liver at the onset of C26 cancer-induced cachexia

**DOI:** 10.1016/j.isci.2025.112030

**Published:** 2025-02-14

**Authors:** Ghita Chaouki, Laurent Parry, Cyrielle Vituret, Céline Jousse, Martin Leremboure, Céline Bourgne, Laurent Mosoni, Yoann Delorme, Mehdi Djelloul-Mazouz, Julien Hermet, Julien Averous, Alain Bruhat, Lydie Combaret, Daniel Taillandier, Isabelle Papet, Laure B. Bindels, Pierre Fafournoux, Anne-Catherine Maurin

**Affiliations:** 1 Unité de Nutrition Humaine, INRAE, Université Clermont Auvergne, UMR 1019, F-63000 Clermont-Ferrand, France; 2 Université Clermont Auvergne, Clermont Auvergne INP, CNRS, Institut de Chimie de Clermont-Ferrand (ICCF), 63000 Clermont-Ferrand, France; 3 Digital PCR Platform Facility of the CHU of Clermont-Ferrand, 63000 Clermont-Ferrand, France; 4 Metabolism and Nutrition Research Group, Louvain Drug Research Institute, Université catholique de Louvain, UCLouvain, Brussels, Belgium; 5 Welbio Department, WEL Research Institute, Wavre, Belgium

**Keywords:** Molecular biology, Cell biology, Cancer

## Abstract

The sequence of events associated with cancer cachexia induction needs to be further characterized. Using the C26 mouse model, we found that prior to cachexia, cancer progression was associated with increased levels of IL-6 and growth differentiation factor 15 (GDF15), highly induced production of positive acute phase proteins (APPs) and reduced levels of most amino acids in the systemic circulation, while signal transducer and activator of transcription 3 (STAT3) signaling was induced (1) in the growing spleen, alongside activation of ribosomal protein S6 (rpS6) and alpha subunit of eukaryotic translation initiation factor-2 (eIF2α) signalings, and (2) in the liver, alongside increased positive-APP expression, decreased *albumin* expression, and upregulation of autophagy. At the onset of cachexia, rpS6 and eIF2α signalings were concomitantly activated in the liver, with increased expression of activating transcription factor 4 (ATF4) target genes involved in amino acid synthesis and transport, as well as autophagy. Data show that pre-cachectic (pre-Cx) alterations in protein/aa homeostasis are followed by activation of eIF2α signaling in the liver, an adaptive mechanism likely regulating protein/amino acid metabolism upon progression to cachexia.

## Highlights

• The production of positive APPs at the pre-Cx stage was as high as at the Cx stage
• Circulating levels of most amino acids were reduced as early as the pre-Cx stage
• EIF2α and rpS6 signalings were concomitantly activated in the liver at the onset of Cx
• ATF4 target genes promoting amino acid availability were overexpressed in Cx liver

## Graphical abstract

**Figure:**
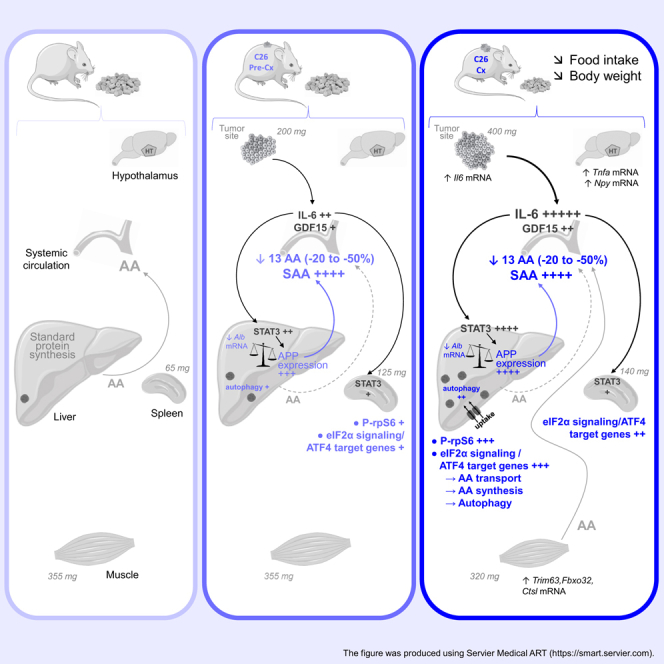

## Introduction

A large proportion of cancer patients suffer from cachexia, a systemic wasting syndrome accelerating the deterioration of health. Cancer cachexia is characterized by a marked involuntary weight loss resulting from a combination of anorexia and atrophy of adipose and skeletal muscle tissues.^1^^,^^2^^,^^3^^,^^4^ This represents one of the most detrimental side effects of cancer as advancing cachexia leads to functional impairments and a general weakness state that reduces tolerance and response to anticancer treatments. This syndrome can be considered as an immuno-metabolic imbalance resulting from neoplastic aggression. Indeed, the increased need of energy and building substrates due to tumor growth and activation of the immune system rapidly leads to systemic inflammation and major metabolic changes.^4^^,^^5^^,^^6^ Despite these needs, marked anorexia most often appears during the pre-cachectic (pre-Cx) phase or in the early stages of cachexia, contributing significantly to body weight loss and the cachexia syndrome.^3^^,^^5^

Previous studies have characterized cancer cachexia as an increasingly perturbed state associated with inflammatory, metabolic and hormonal changes leading to alterations in behavior and body composition.^5^ Progression to the cachectic (Cx) state involves both reciprocal effects between the tumor and host tissues and interactions between several tissues and organs (liver, adipose tissue, muscle, etc.).^5^^,^^7^^,^^8^^,^^9^ Studies have highlighted the central role of some pro-inflammatory and stress cytokines in metabolic and food intake alterations during cachexia. These include tumor necrosis factor-α (TNF-α), interleukin (IL)-6, IL-1β, and growth differentiation factor 15 (GDF15),^8^^,^^10^^,^^11^^,^^12^^,^^13^^,^^14^ whose release is progressively increased from the tumor site as the tumor grows. Particularly, IL-6 and GDF15 have been functionally involved in animal models of cancer anorexia-cachexia.^15^^,^^16^^,^^17^^,^^18^^,^^19^^,^^20^^,^^21^^,^^22^ Nevertheless, the factors and events associated with the pre-Cx phase (before any weight loss) and transition to cachexia need to be better characterized.

During disease, the activity of many cell types is altered by a state of stress that may arise from the presence of foreign components, the influx of stress mediators such as cytokines, or energy and nutrient deprivation. One of the mechanisms enabling the cell to cope with stress is the Integrated Stress Response, controlled by the phosphorylation of the alpha subunit of eukaryotic translation initiation factor-2 (eIF2α). eIF2α phosphorylation triggers both a reduction in protein synthesis in order to save resources and an adaptive gene expression program at the transcriptional level, through upregulated expression of activating transcription factor 4 (ATF4).^23^^,^^24^^,^^25^^,^^26^ ATF4 notably regulates the expression of many genes involved in amino acid metabolism and responses to mitochondrial- and endoplasmic reticulum stresses.^24^^,^^25^^,^^26^^,^^27^^,^^28^^,^^29^^,^^30^ Among the four kinases able to phosphorylate eIF2α in response to different kinds of stress, the general control non-derepressible 2 (GCN2) kinase (also known as Eif2ak4) is specifically activated by amino acid scarcities. Previous studies have highlighted the central role of the GCN2-eIF2α-ATF4 pathway in early food intake reduction response to nutritional stresses of one essential amino acid deficiency in the meal.^31^^,^^32^^,^^33^^,^^34^ Furthermore, this signaling pathway has also been involved in upregulation of autophagy during amino acid deprivation in different cell types.^29^^,^^35^^,^^36^^,^^37^

In this study, our aim was to characterize early events resulting from tumor growth and preceding and/or being associated with the onset of the cachexia syndrome. For that purpose, we used the colon-26 adenocarcinoma (C26) mouse model of cancer cachexia^38^ in which anorexia has been described as preceding or being concomitant with body weight loss,^19^^,^^39^ as in humans.^3^^,^^5^ At the pre-Cx stage, we found that the relatively moderate increase in IL-6 was associated with activation of signal transducer and activator of transcription 3 (STAT3) signaling in the spleen and liver, while in the systemic circulation, positive acute phase proteins (APPs) (serum amyloid A [SAA]) production was already very high and levels of most amino acids were markedly reduced. Furthermore, the data showed simultaneous activation of ribosomal protein S6 (rpS6) and eIF2α signalings and increased expression of ATF4 target genes involved in amino acid supply (1) in the spleen as early as the pre-Cx stage, and (2) in the liver at the onset of cachexia, suggesting upregulation of protein/amino acid metabolism in these organs early during cancer progression.

## Results

### Characterization of the pre-Cx and Cx stages

**Figure 1:**
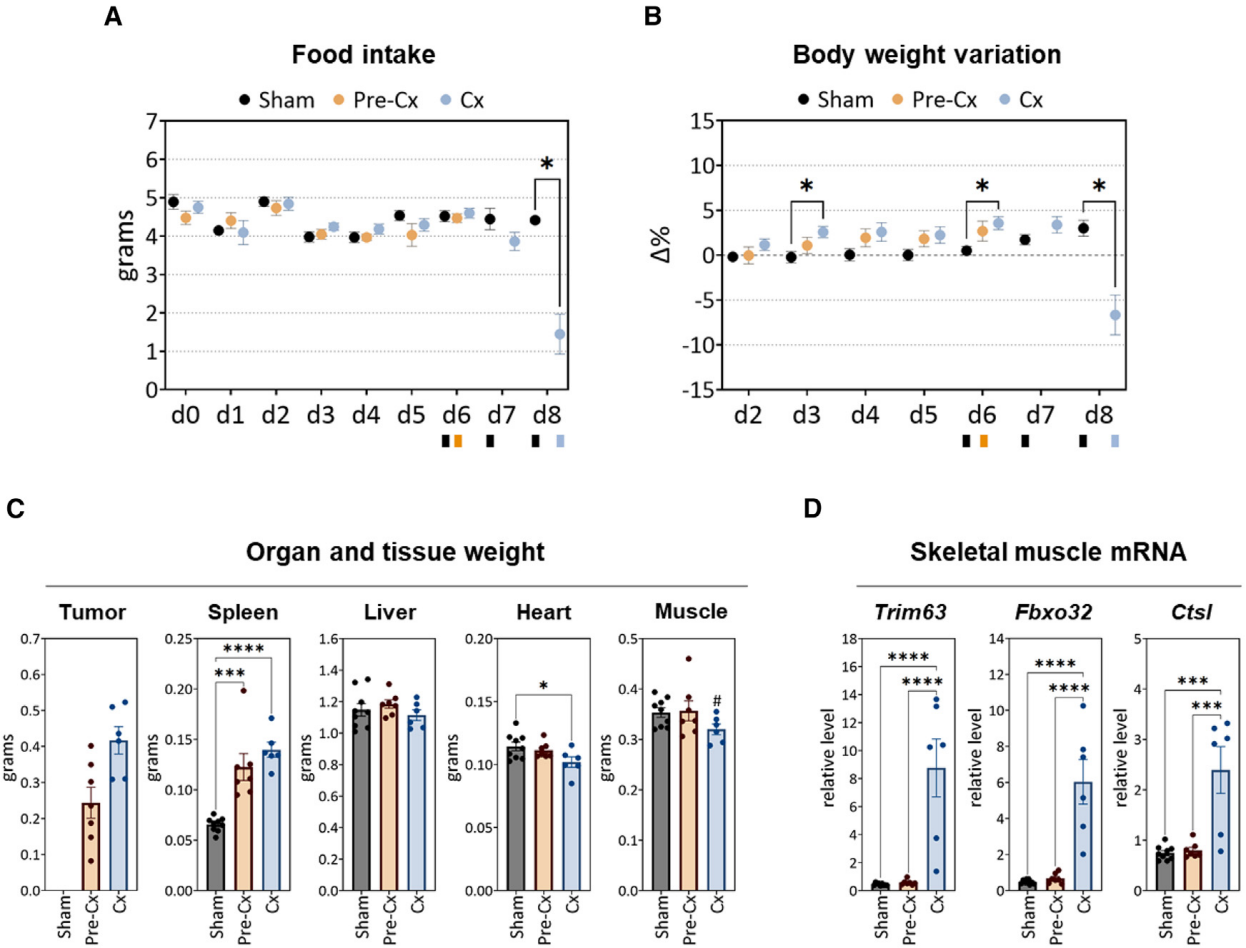
Unlike pre-Cx mice, Cx mice were characterized by reduced food intake, body weight, and muscle mass, as well as increased markers of skeletal muscle atrophy At day 0, mice received either an implantation of 10^6^ C26 cells (C26 tumor-bearing mice) or an injection of NaCl (sham-injected mice). C26 mice were necropsied either at day 6 (pre-Cx group) or day 8 (Cx group) and organs and tissues were weighed and samples harvested. The sham-injected group combined control mice obtained on days 6, 7, and 8 post-injection. Data are represented as mean ± SEM. (A) Kinetic analysis of food intake. Food intake was measured every 24 h from the time point of implantation of C26 cells (day 0). (B) Kinetic analysis of body weight. Body weight was measured every 24 h and expressed as a delta-percentage of reference values as measured at day 0. (C) Organ and tissue weights at the time of sacrifice. Boxes below the graphs in (A) and (B) represent the sacrifice time points according to the different groups. Regarding skeletal muscle, the average weight of the set of gastrocnemius plus plantaris plus soleus is shown. (D) Relative mRNA levels of atrophy-related genes in skeletal muscle (as compared to *Ywhaz* mRNA level). To assess the significance of differences in food intake and body weight between groups over time, two-way analyses of variance (ANOVA) were performed for each of these parameters and completed by Tukey’s multiple comparison tests to analyze differences between groups at each time point. The first ANOVA, focused on data obtained up to day 6 for the three groups (sham, pre-Cx, and Cx), highlighted an overall significant effect of time (*p* < 0.0001), but no difference between groups neither for food intake or body weight. The second ANOVA took into account the sham-injected- and Cx groups from day 0 to day 8. Daily food intake was found to vary significantly according both to the presence of the tumor (*p* = 0.0151) and time (*p* < 0.0001), with a significant interaction between the two parameters (*p* < 0.0001). Analysis of the overall body weight data highlighted a significant effect of time on this parameter (*p* = 0.0082) as well as a significant interaction between the presence of the tumor and time (*p* < 0.0001). For other analyses (C and D), the significance of differences between groups was assessed by one-way ANOVA followed by Tukey’s multiple comparisons. (∗*p* < 0.05; ∗∗∗*p* < 0.001; ∗∗∗∗*p* < 0.0001). In (C), the significance of the difference between the muscle weights of the Cx- and sham-injected groups was also assessed by direct pairwise comparison (#*p* < 0.05, t test). See also Figure S1.

A preliminary experiment enabled us to determine the kinetics of variation in food intake and body weight after C26 cell implantation (day 0), and to specifically define day 6 and day 8 post-implantation as corresponding to pre-Cx and early Cx stages, respectively (data not shown). We then carried out a second experiment to collect samples and analyze biological parameters from C26 mice at the pre-Cx or Cx stage, as well as from sham-injected mice. Food intake and body weight were measured daily from the day of implantation of C26 cells. On the 8th day, we observed a significant reduction in food intake of C26 mice compared to sham-injected mice (Figure 1A), associated with a decreased body weight (Figure 1B). At the pre-Cx stage, tumor weight was still modest (0.24 g or less than 1% of body weight) and it was increased by 1.7-fold at the Cx stage (Figure 1C). Spleen weight in pre-Cx mice was almost twice that of sham-injected mice, and did not increase further in the Cx group, while liver weight was similar in all groups. Heart weight was lower in the C26-Cx group than in the sham-injected group (Figure 1C). The weight of skeletal muscle, as assessed for the gastrocnemius plus soleus plus plantaris set, was found to not differ between the three groups following ANOVA; however, direct pairwise comparison between C26-Cx mice and sham-injected mice revealed reduced muscle weight in the C26-Cx group (around −10%; #*p* = 0.038, t test) (Figure 1C). Moreover, mRNA analyses in skeletal muscle confirmed an increased expression of the protein breakdown-related genes^40^^,^^41^^,^^42^^,^^43^ *Trim63* and *Fbxo32* (encoding ubiquitin-protein ligases also known as MuRF1 and MAFbx, respectively), and *Ctsl* (encoding the cysteine protease cathepsin L) in the C26-Cx group, but not in the C26-pre-Cx group (Figure 1D). Thus, unlike the pre-Cx group, the Cx group was characterized by reduced food intake, body weight, and muscle mass, as well as increased expression of atrophy-related genes in skeletal muscle.

To further characterize early events linked to the onset of anorexia-cachexia, we analyzed the expression of neuropeptides involved in food intake control in the hypothalamus. We found that expression level of *Pomc*, encoding the anorectic neuropeptide pro-opiomelanocortin-alpha was unaltered, as measured at whole hypothalamus level, regardless of the group (see Figure S1A). By contrast, mRNA level of *Npy*, encoding the orexigenic neuropeptide Y, was 2-fold higher in the Cx group compared to sham-injected- and pre-Cx groups. This result supports previous data obtained with several animal models of cancer cachexia.^44^^,^^45^^,^^46^^,^^47^^,^^48^^,^^49^ This could reflect an attempt of the organism to increase nutrient supply by using physiological mechanisms promoting food intake. In addition, we observed that mRNA level of the pro-inflammatory cytokine TNF-α was doubled in the hypothalamus of Cx mice compared to sham-injected- and pre-Cx mice, while mRNA levels of IL-1β and IL-6 remained unchanged (see Figure S1B). Thus, the onset of anorexia-cachexia was associated with an increased hypothalamic expression of *Tnfa* and *Npy*. However, we did not detect any variation of expression of these mRNAs in the hypothalamus of C26 pre-Cx mice compared to sham-injected animals (see Figure S1).

### Increased production of IL-6 and GDF15 and reduced levels of most amino acids in systemic circulation as early as the pre-Cx stage

**Figure 2:**
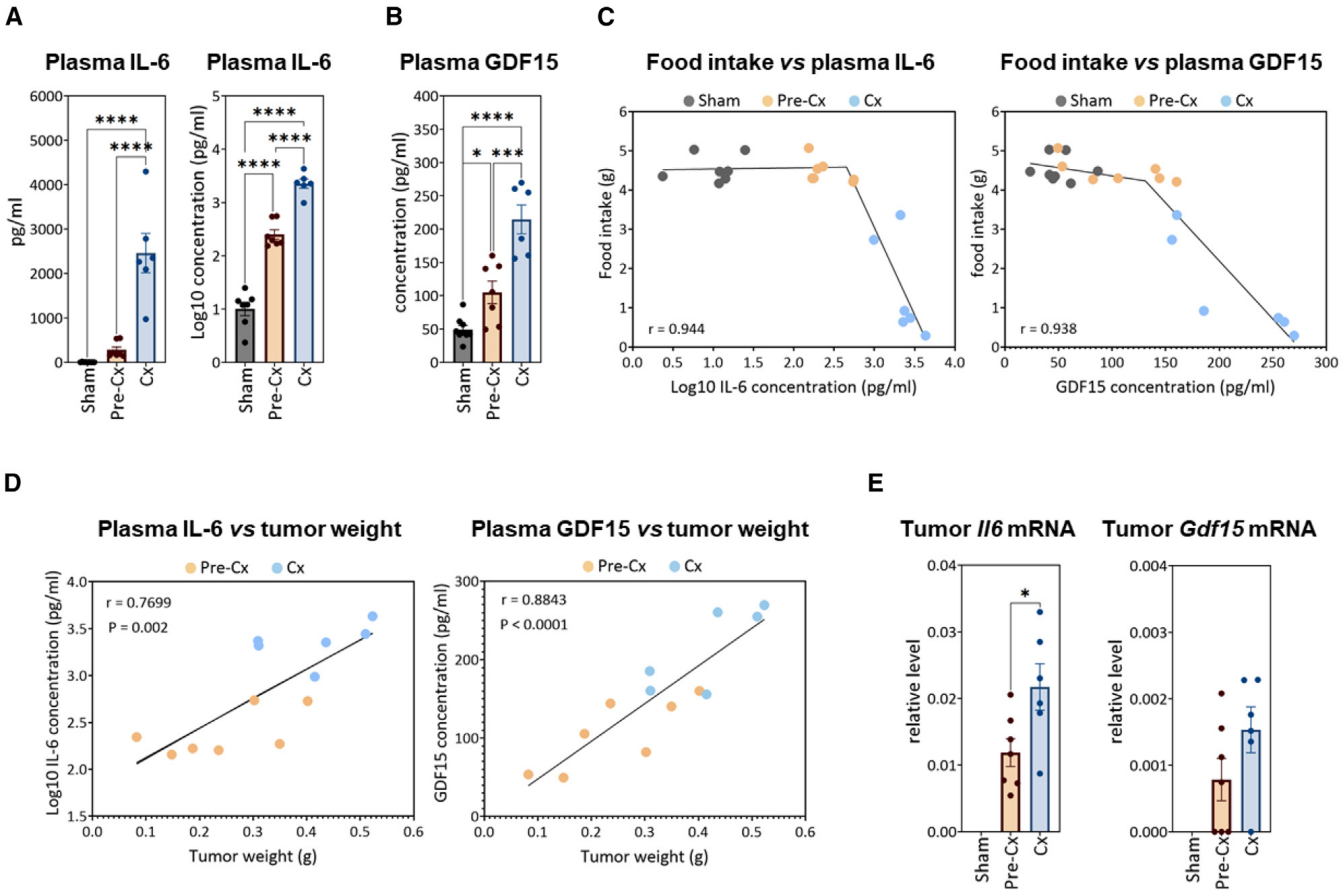
Comparative regulation of the cachectic cytokines IL-6 and GDF15 at pre-Cx and Cx stages (A) Systemic circulating levels of IL-6 (real and log-transformed values). (B) Systemic circulating levels of GDF15. (C) Inverse relationship between plasma concentration of either IL-6 or GDF15 and food intake level. Data analysis using a non-linear regression test revealed a segmental linear relationship and a threshold effect (anorexia was associated with plasma GDF15 levels at least above 131 pg/mL and plasma IL-6 levels at least above log10 (2.653) pg/mL ≈ 450 pg/mL). (D) Positive linear relationship between tumor weight and circulating IL-6 or GDF15 levels. The significance of the correlation between the two parameters was assessed by the Pearson’s test. (E) Relative mRNA levels of *Il-6* and *Gdf15* at the tumor site, as determined by digital qPCR (relative to *Ppia* mRNA level). In (A), (B), and (E), data are represented as mean ± SEM. The significance of differences between groups in (A), (B), and (E) was assessed by one-way ANOVA followed by Tukey’s multiple comparisons (∗*p* < 0.05; ∗∗∗*p* < 0.001; ∗∗∗∗*p* < 0.0001). See also Figure S2.

Both IL-6 and GDF15 have been functionally involved in cancer anorexia-cachexia.^15^^,^^16^^,^^17^^,^^18^^,^^19^^,^^20^^,^^21^^,^^22^ Consistently, high plasma levels of both cytokines have been associated with cachexia in the C26 model.^18^^,^^19^^,^^20^^,^^50^^,^^51^ We therefore measured circulating levels of these previously characterized critical factors to help contextualize our model in the progression from the pre-Cx to Cx stage. While IL-6 was almost undetectable in the sham-injected group, its mean concentration was 289 pg/mL in pre-Cx mice and reached 2,469 pg/mL in Cx mice (Figure 2A). As IL-6 concentration values followed a lognormal distribution, we performed a logarithmic transformation, which significantly increased the probability of a normal Gaussian distribution and highlighted that IL-6 concentration log-transformed values were significantly increased in pre-Cx- vs. sham-injected mice and further elevated in Cx- vs. pre-Cx mice (Figure 2A). We also found that circulating GDF15 levels were gradually increased from 49 pg/mL in the sham-injected group to 105 pg/mL and 214 pg/mL in the pre-Cx- and Cx groups, respectively (Figure 2B). Thus, despite increased plasma levels of IL-6 and GDF15 at the pre-Cx stage, food intake was unaffected. Consistently, analysis of the relationship between plasma concentration of these cytokines and extent of food intake inhibition revealed a segmental profile with two linear regression segments (Figure 2C). According to these models, anorexia was associated with GDF15 and IL-6 concentrations above at least 131 pg/mL and 450 pg/mL, respectively. We also observed that both GDF15 and log-transformed IL-6 concentration values positively and linearly correlated with tumor weight (Figure 2D). Furthermore, *Il-6* mRNA expression at the tumor site was increased 2-fold in Cx- compared to pre-Cx mice, while *Gdf15* mRNA level remained unchanged (Figure 2E). In addition, mRNA levels of both cytokines were unaffected in peripheral tissues such as liver and intestine, at either the pre-Cx- or Cx stage (see Figure S2). Overall, these data show that tumor growth led to a significant increase in circulating levels of IL-6 and GDF15 before the onset of anorexia-cachexia, the latter being associated with plasma concentrations of these two cytokines above threshold values.

**Figure 3:**
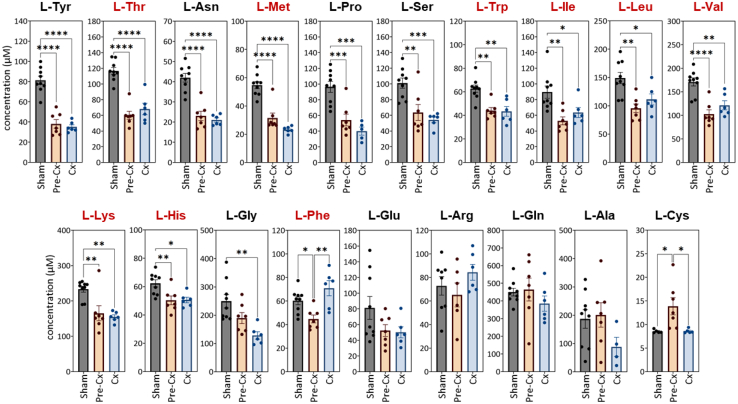
Marked reduction in the level of most amino acids in the systemic circulation as early as the pre-Cx stage Blood was collected at the time of sacrifice by intra-cardiac puncture. Concentrations of the different amino acids were determined from plasma samples. Data are represented as mean ± SEM. The significance of differences between groups was assessed by one-way ANOVA followed by Tukey’s multiple comparisons (∗*p* < 0.05; ∗∗*p* < 0.01; ∗∗∗*p* < 0.001; ∗∗∗∗*p* < 0.0001). Essential amino acids are indicated in red.

Next, since amino acid-homeostasis and -blood concentrations may be affected by neoplastic aggression, we measured amino acid levels in systemic circulation. Compared to sham-injected animals, C26 mice had reduced plasma concentrations of thirteen L-amino acids as early as the pre-Cx stage: tyrosine, threonine, asparagine, methionine, proline, serine, tryptophan, isoleucine, leucine, valine, lysine, histidine, and phenylalanine (Figure 3). For almost all these amino acids, this effect was also observed, with the same amplitude, in the Cx group. Interestingly, phenylalanine was the only amino acid whose level was higher at the Cx stage compared to the pre-Cx stage. The pre-Cx stage-related reduction concerned all essential amino acids, as well as some non-essential ones (Figure 3). The magnitude of the decrease ranged from 40% to 60% for tyrosine, threonine, asparagine, methionine, proline, and serine. Glycine tended to show the same profile, with a significant decrease at the Cx stage only (Figure 3). Cysteine was the only amino acid whose circulating level was higher in C26 mice than in sham-injected animals, and only at the pre-Cx stage. Finally, we did not observe any significant change in plasma concentration of glutamate, arginine, glutamine, and alanine. Overall, amino acid profiles in the systemic circulation were strongly affected at the pre-Cx stage, while food intake was unaffected.

During cachexia, it is recognized that the activation of muscle protein degradation aims to mobilize amino acids for transfer as substrates to the tumor and visceral organs.^6^^,^^52^^,^^53^^,^^54^ Recourse to this deleterious process demonstrates the magnitude of the need, and suggests that reductions in circulating amino acid levels as early as the pre-Cx stage may reflect an early increase in amino acid utilization at these sites.

### Activation of signaling pathways regulating protein/amino acid homeostasis in the pre-Cx spleen

In C26 mice, spleen weight was increased by 2-fold as early as the pre-Cx stage (Figure 1C). This observation was consistent with data from literature indicating splenomegaly in C26 cancer-related cachexia.^18^^,^^55^ As suggested elsewhere, this was likely to be the result of hyperfunction and immune hyperplasia, which could represent a significant consumption of amino acids.^56^

**Figure 4:**
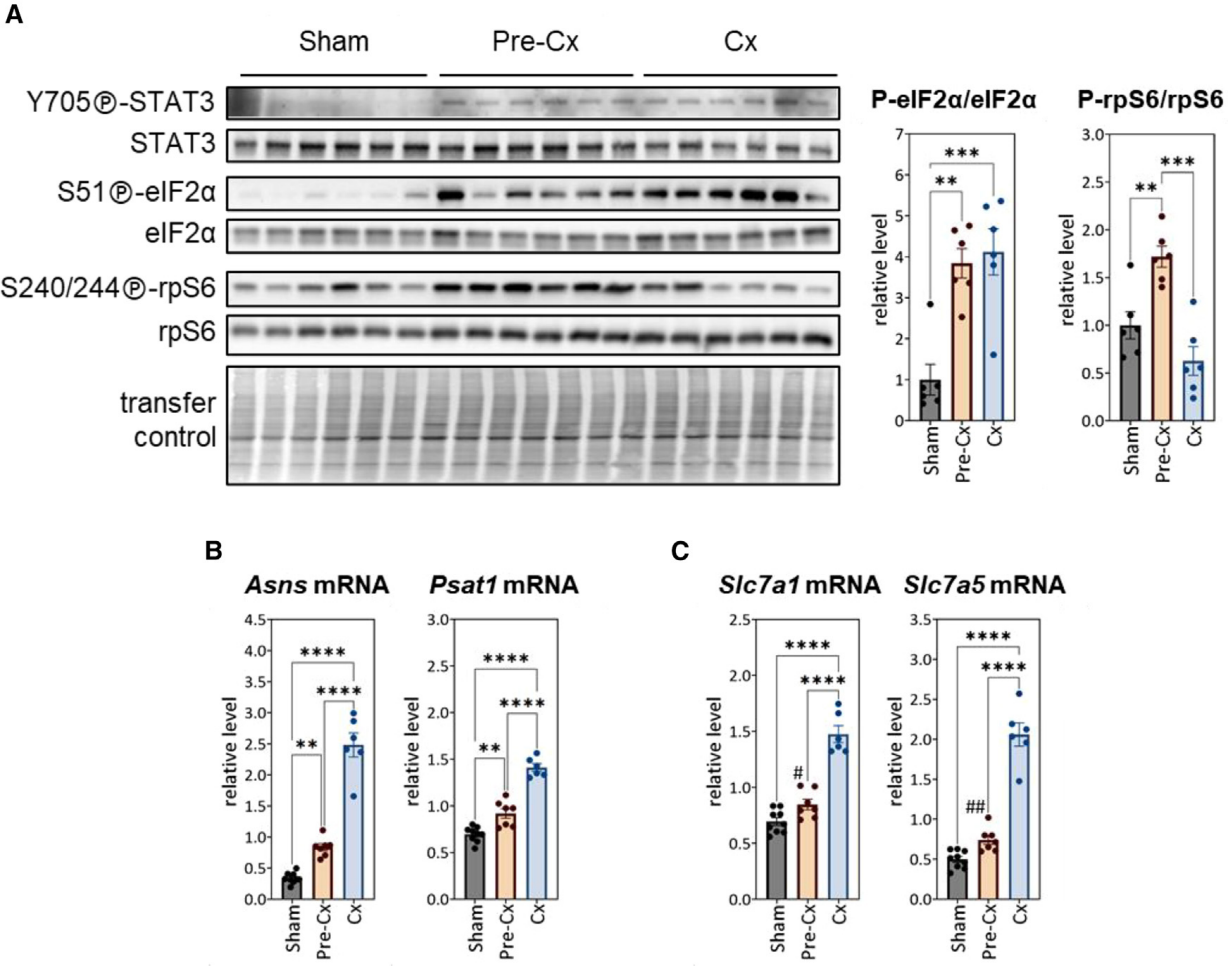
Activation of molecular pathways related to inflammatory cytokine signaling and regulation of protein/amino acid metabolism in the pre-Cxspleen (A) Analysis of [Y705]-STAT3, (S51)-eIF2α and (S240/244)-rpS6 phosphorylation by western-blot. TGX signals were used to control protein transfer efficiency. For each mouse, the calculation of the ratio between the signal intensity of the phosphorylated form and the signal intensity of the total form was performed. The significance of differences between groups was assessed by one-way ANOVA followed by Tukey’s multiple comparisons (∗∗*p* < 0.01; ∗∗∗*p* < 0.001). Data are represented as mean ± SEM. (B and C) mRNA levels of ATF4-target genes involved in amino acid biosynthesis (B) and amino acid transport (C) expressed relative to *Ppia* mRNA level. The significance of differences between groups was assessed by one-way ANOVA followed by Tukey’s multiple comparisons (∗∗*p* < 0.01; ∗∗∗∗*p* < 0.0001). In (C), the significance of differences between the relative mRNA levels in the pre-Cx- and sham-injected groups was also assessed by direct pairwise comparison (#*p* = 0.018 and ##*p* = 0.002 by t test). Data are represented as mean ± SEM.

We first analyzed spleen responsiveness to inflammatory stimuli. Mechanistically, IL-6 has been shown to act mainly through activating STAT3.^56^^,^^57^^,^^58^^,^^59^^,^^60^ We observed that (Tyr705)-STAT3 phosphorylation was induced in the spleen of C26 mice as early as the pre-Cx stage (Figure 4A), indicating activated state of this effector,^60^^,^^61^ despite a rather moderate increase in circulating level of IL-6 at this stage (Figure 2A).

Then, we analyzed the activity of the two major signaling pathways involved in protein/amino acid homeostasis regulation, namely mechanistic target of rapamycin complex 1 (mTORC1)- and eIF2α-ATF4 signaling pathways, at the whole spleen level. mTORC1 kinase complex plays a key role in sensing amino acid availability and promoting protein synthesis accordingly, by notably activating rpS6 kinase (S6K1).^62^ We examined the activity of S6K1, which reflects the impact of mTORC1 on protein synthesis, by analyzing the phosphorylation level of its substrate, rpS6. Interestingly, phosphorylation of (S240/244)-rpS6 was transiently upregulated in the spleen of C26 mice, at the pre-Cx stage only. The eIF2α-ATF4 signaling pathway is an important regulator of protein/amino acid homeostasis involved in cell adaptation to stress situations, by inhibiting translation and triggering an ATF4-mediated gene expression program including many genes involved in amino acid metabolism.^24^^,^^25^^,^^26^^,^^30^^,^^63^ We observed that eIF2α signaling was activated in the spleen of C26 mice at both the pre-Cx and Cx stages, as evidenced by increased phosphorylation of eIF2α on Ser51 residue. Consistently, expression level of ATF4 target genes involved in amino acid synthesis (i.e., *Asns* and *Psat1*, involved in asparagine and serine synthesis, respectively) (Figure 4B) and amino acid transport (i.e., *Slc7a1* and *Slc7a5*, encoding CAT-1 and LAT1 plasma membrane amino acid transporters, respectively) (Figure 4C) was increased at the pre-Cx stage, an effect that was clearly enhanced at the Cx stage.

Thus, alongside increased spleen size at the pre-Cx stage, signaling pathways regulating amino acid availability and utilization were activated in this organ, probably to cope with elevated rates of protein and nucleotide synthesis linked to tumor-induced immune cell proliferation.

### Strong induction of positive-APP production as early as the pre-Cx stage

**Figure 5:**
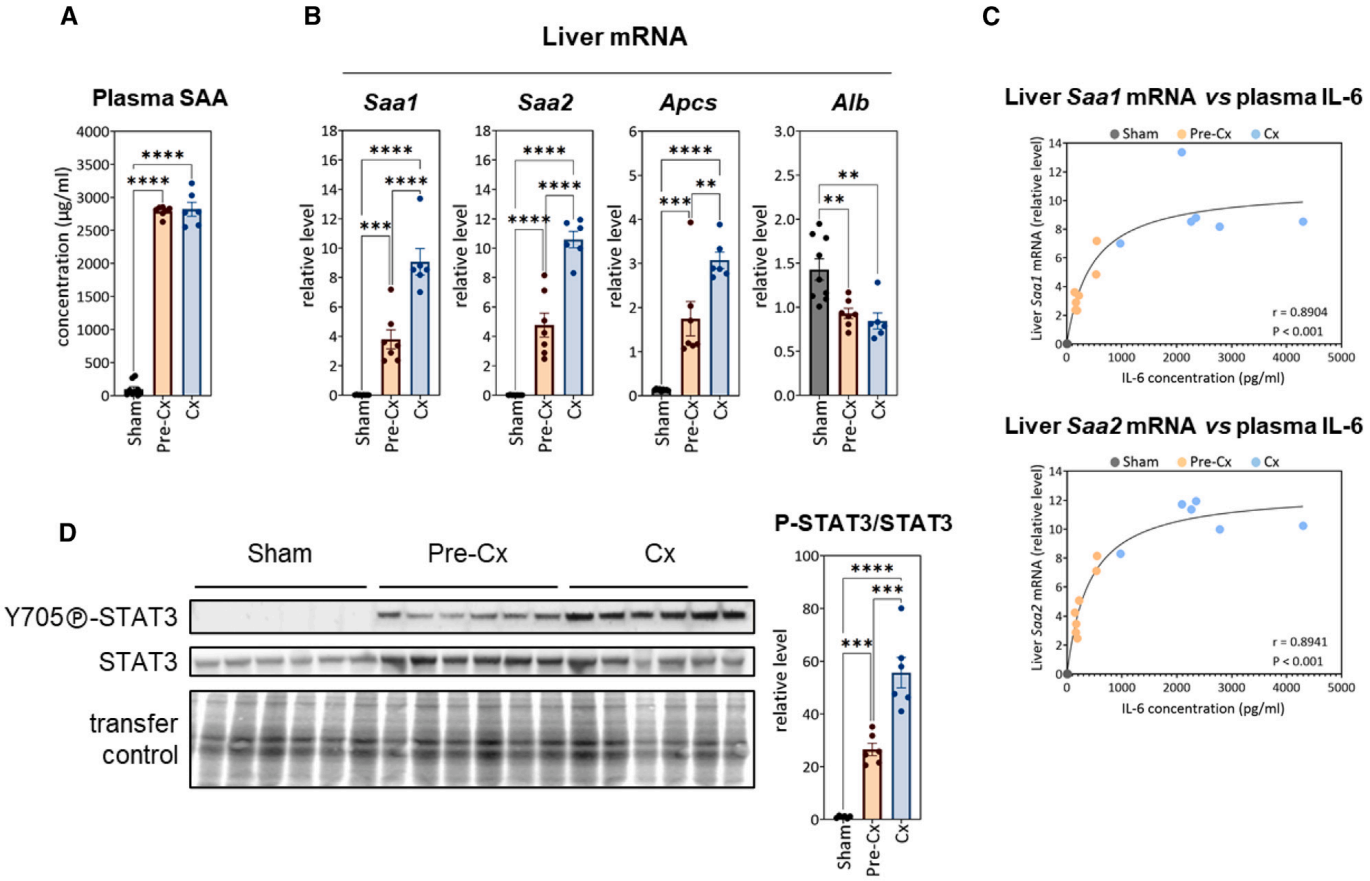
Huge induction of positive-APP production as early as the pre-Cx stage (A) Systemic circulating levels of SAAs. (B) Relative mRNA levels of *Saa1*, *Saa2*, *Apcs*, and *Alb* in the liver (as compared to *Ppia* mRNA level). (C) Strong correlation between IL-6 circulating levels and SAA gene expression in the liver of C26 mice. A Spearman correlation test was performed. (D) Western-blot analysis of (Tyr705)-STAT3 phosphorylation. For each mouse, the calculation of the ratio between the signal intensity of the phosphorylated form and the signal intensity of the total form was performed. TGX signals were used to control protein transfer efficiency. In (A), (B), and (D), data are represented as mean ± SEM, and the significance of differences between groups was assessed by one-way ANOVA followed by Tukey’s multiple comparisons (∗∗*p* < 0.01; ∗∗∗*p* < 0.001; ∗∗∗∗*p* < 0.0001).

In addition to supporting active cell proliferation in spleen and tumor, increased amino acid requirements may result from highly induced synthesis of exported proteins specifically involved in response to acute inflammation. APPs are blood proteins highly produced as part of the acute phase response (APR), an early defense and protection system activated during various types of aggression.^64^^,^^65^ Members of the SAA family, major positive APPs in mice and humans, are extensively produced during acute inflammation.^64^^,^^65^ We found that SAA levels were dramatically increased in the systemic circulation in C26 mice as early as the pre-Cx stage (mean concentration 2.79 mg/mL), an effect that was not amplified at the Cx stage (mean concentration 2.82 mg/mL) (Figure 5A).

Hepatocytes are considered the main source of APPs.^66^ In liver, we observed that expression of *Saa1*, *Saa2*, and *Apcs*, encoding three major positive APPs (two SAA isoforms and the serum amyloid P component, respectively) was strongly induced at the pre-Cx stage, and further increased at the Cx stage (Figure 5B). As IL-6 has been directly involved in upregulation of APP expression,^67^^,^^68^^,^^69^^,^^70^ we plotted hepatic *Saa1* and *Saa2* mRNA levels as a function of circulating IL-6 concentration for each animal (i.e., data given in Figure 2A), an analysis that revealed a strong correlation between the two parameters (Figure 5C). Conversely, mRNA level of albumin, a negative APP, was decreased by 35% as early as the pre-Cx stage (Figure 5B). Mechanistically, IL-6 has been shown to regulate positive-APP gene expression mainly through activating STAT3 signaling,^56^^,^^57^^,^^58^^,^^59^^,^^71^^,^^72^ and previous work has evidenced STAT3 signaling activation in the liver of Cx C26 mice.^73^ Consistently, we observed that (Tyr705)-STAT3 phosphorylation was clearly induced in the liver of C26 mice as early as the pre-Cx stage, indicating activated state of this transcription factor (Figure 5D). Thus, even before the onset of cachexia, cancer progression elicited a huge induction of positive-APP production, while *albumin* expression was reduced.

### Upregulation of mechanisms promoting amino acid availability and utilization in the liver at the transition from the pre-Cx to Cx stage

The liver plays a central role in the regulation of protein/amino acid homeostasis, releasing many amino acids used by other organs and tissues,^74^ and producing the majority of proteins exported to plasma.^75^ We therefore carried out additional analyses to investigate parameters related to amino acid utilization and availability in the liver.

**Figure 6:**
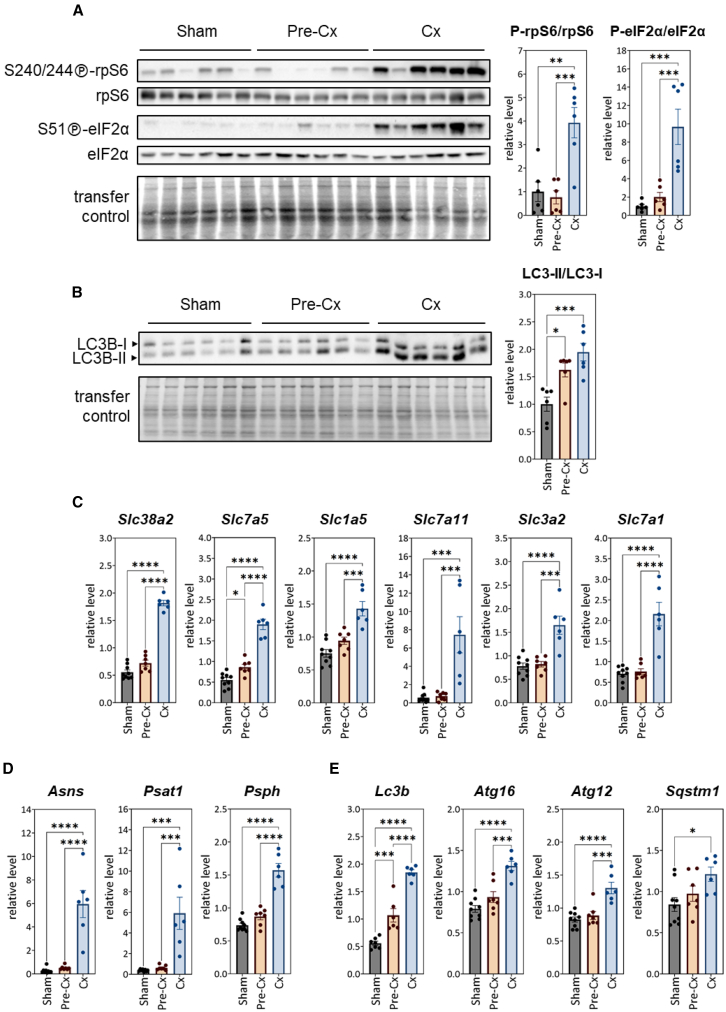
Upregulation of mechanisms promoting amino acid availability and utilization in the liver during the transition from pre-Cx to Cx stage (A) Western-blot analysis of (S240/244)-rpS6 phosphorylation and (S51)-eIF2α phosphorylation. For each mouse, the ratio between the signal intensities obtained for the phosphorylated form and the total form, respectively, was calculated. (B) Western-blot analysis of LC3B conversion. For each mouse, the ratio between the signal intensities obtained for the lipidated form anchored in the autophagosome membrane (LC3-II), and for the cytosolic form (LC3-I), respectively, was calculated. Increased ratio was an indicator of new autophagosomes recruitment. In (A) and (B), TGX signals were used to control protein transfer efficiency. (C–E) mRNA levels of ATF4-target genes involved in amino acid transport (C), amino acid biosynthesis (D), and autophagy (E), expressed relative to *Ppia* mRNA level. All data are represented as mean ± SEM. The significance of differences between groups was assessed by one-way ANOVA followed by Tukey’s multiple comparisons (∗*p* < 0.05; ∗∗*p* < 0.01; ∗∗∗*p* < 0.001; ∗∗∗∗*p* < 0.0001). See also Figure S3.

We first observed that (S240/244)-rpS6 phosphorylation was unaffected at the pre-Cx stage, but strongly increased in livers of Cx mice compared to sham-injected- and pre-Cx mice (Figure 6A), suggesting an increase in mTORC1/S6K1-mediated hepatic protein synthesis at the onset of cachexia. Then, as autophagy is a degradation process that can provide substantial amounts of amino acids through lysis of endogenous liver proteins,^76^^,^^77^^,^^78^ we analyzed the lipidation of MAP1LC3B protein (microtubule-associated protein 1 light chain 3 beta, LC3B) as a major marker of autophagosome formation by western blotting. The signal ratio between the lipid-bound LC3B-II form and the cytosolic LC3B-I form increased at pre-Cx and Cx stages, indicating upregulation of autophagy (Figure 6B). mTORC1 regulates autophagy.^62^ By notably phosphorylating the autophagy-initiating serine/threonine-protein kinase Unc-51-like kinase 1 (ULK1) on Ser757 residue, mTORC1 inhibits autophagy when amino acids are available.^79^^,^^80^^,^^81^^,^^82^ Western blot analysis of liver protein extracts showed that intensity of the phospho-(Ser757)-ULK1 signal varied within groups (see Figure S3). However, the signal intensity ratio between the phosphorylated and total forms (phospho-(Ser757)-ULK1/ULK1) was lower in the pre-Cx group than in the sham-injected group. Moreover, this decrease was more an effect of increased total ULK1 signal (see Figure S3), a parameter that has already been considered as an indicator of upregulated autophagy in previous studies.^83^^,^^84^ On the whole, these results support an upregulation of autophagy at the pre-Cx stage.

We then analyzed the eIF2α-ATF4 signaling pathway as a major regulator of protein/amino acid homeostasis.^24^^,^^25^^,^^26^ In addition to inhibiting translation, eIF2α phosphorylation has been shown to trigger an ATF4-mediated gene expression program including many genes involved in amino acid metabolism.^24^^,^^25^^,^^26^^,^^29^^,^^30^^,^^63^ We found that eIF2α phosphorylation was not affected at the pre-Cx stage but was clearly induced in the liver of Cx mice (Figure 6A). Consistently, expression levels of the ATF4-target genes *Slc38a2*, *Slc7a5*, *Slc1a5*, *Slc7a11*, *Slc3a2*, and *Slc7a1*, encoding several plasma membrane amino acid transporters,^27^^,^^30^^,^^85^^,^^86^^,^^87^ were markedly increased in the liver of C26-Cx mice compared to sham-injected and pre-Cx mice (Figure 6C). Moreover, mRNA levels of ATF4 target genes involved in amino acid synthesis^27^^,^^30^^,^^88^ (i.e., *Asns*, *Psat1*, and *Psph*) (Figure 6D) and autophagy^29^ (i.e., *Lc3b*, *Atg16l1*, *Atg12*, and *Sqstm1*) (Figure 6E) were also clearly upregulated in livers of C26-Cx mice. Interestingly, relative expression levels of *Asns* and *Psat1*, involved in asparagine and serine synthesis, were increased 22- and 15-fold, respectively, in livers of Cx mice compared to sham-injected animals (Figure 6D). These results suggest that ATF4 may contribute to promoting the availability of amino acids in the liver from the onset of cachexia, by upregulating the expression of genes involved in amino acid biosynthesis and amino acid transport, as well as autophagy, at a time when high phosphorylation of rpS6 likely indicates an mTORC1-mediated increase in liver protein synthesis.

## Discussion

In this study, we examined events associated with the pre-Cx stage and early cachexia during cancer progression, and related to protein/amino acid metabolism. We found that in systemic circulation, pre-Cx cancer progression was associated with moderate increases in IL-6 and GDF15 concentrations, dramatic induction of positive-APP production and marked reductions in the levels of most amino acids. STAT3 signaling was activated in the spleen and liver as early as the pre-Cx stage. This was associated with upregulated rpS6 and eIF2α signalings in the spleen, coinciding with an early and marked increase in the size of this organ. In the liver, an increase in the expression of positive-APPs, a decrease in the expression of albumin and an upregulation of autophagy occurred at the pre-Cx phase, while at the onset of cachexia, rpS6 phosphorylation and eIF2α signaling were strongly activated in this organ, along with increased expression of ATF4-target genes involved in amino acid synthesis and transport, as well as autophagy. Thus, neoplastic aggression had a strong impact on amino acid homeostasis even before the onset of cachexia, and progression to cachexia was associated with activation of adaptive mechanisms involved in regulating amino acid availability and utilization in the spleen and liver, including mTORC1 and eIF2α-ATF4 signaling pathways, presumably to cope with the sharp increase in (1) cell growth and (2) positive-APP production, respectively, in these organs.

We found elevated circulating levels of IL-6 and GDF15 at established cachexia, in agreement with previous studies using the C26 tumor-bearing mouse model,^18^^,^^19^^,^^20^^,^^51^^,^^89^ but also moderately increased levels of these cytokines at the pre-Cx stage. These two cytokines have been functionally involved in cancer cachexia.^15^^,^^16^^,^^17^^,^^18^^,^^19^^,^^20^^,^^21^^,^^22^ According to data from literature, GDF15 and IL-6 are likely produced at the tumor site,^4^^,^^15^^,^^90^ composed of cancer cells as well as various infiltrated cell populations in the tumor microenvironment.^7^^,^^91^ In line with this, we found that levels of *Il-6* mRNA were not affected in peripheral tissues (liver and intestine) of C26 mice, but were almost doubled at the tumor site in the Cx group compared to pre-Cx group. Since tumor weight almost doubled between these two stages, the combined effects of increased expression and increased tumor size could explain the exponential rise in circulating levels of IL-6. *Gdf15* mRNA levels were not altered in peripheral tissues or at the tumor site, even at an early stage of cachexia. Nevertheless, we can speculate that the nearly 2-fold increase in tumor size between the pre-Cx and Cx stages could have led to the observed linear increase in circulating levels of GDF15, even with a stable production rate at the tumor site, as well as to the strong linear correlation between circulating GDF15 levels and tumor weight (r = 0.8843). Importantly, increased circulating levels of IL-6 and GDF15 were observed even before any reduction in food intake, body weight, or muscle mass, and these levels were further elevated at the onset of anorexia-cachexia. Regarding GDF15 anorectic action, such a threshold effect has been suggested in other studies.^92^^,^^93^^,^^94^ Since GDF15 has been shown to perform a number of diverse functions in addition to regulating food intake,^95^ this cytokine was likely to be involved in aspects other than anorexia during pre-cachexia. Regarding IL-6, given that its STAT3-dependent effector signaling was activated at least in liver and spleen at the pre-Cx stage, we can assume that this cytokine induced/initiated, early and at relatively moderate levels, changes in target tissue functions, such as stimulation of hepatic positive-APP production.

We observed that a significant reduction (−25% to −60%) in systemic circulating levels of many amino acids was associated with tumor growth as early as the pre-Cx phase, demonstrating that reduced dietary intake was not involved. Consistently, in a study using the MAC16 transplant mouse model, which does not affect food intake, the authors observed that the Cx state was associated with decreased plasma concentrations of most of the amino acids analyzed.^96^ Furthermore, using the C26 mouse model, previous works have highlighted that cancer-related cachexia results in a unique metabolic imprint distinct from caloric restriction alone, including reduced levels of specific amino acids.^97^^,^^98^ In line with a recent study,^99^ our data support the idea that the decrease in levels of most amino acids in the systemic circulation is an early event that occurs during tumor growth prior to cachexia, and is not primarily related to a reduction in food intake and/or body weight. This could be the result of enhanced amino acid utilization to meet energy and nitrogen substrate requirements associated with increasing tumor and spleen size, as well as increased production of immune-related proteins. Indeed, over the course of cachexia, it is recognized that amino acids are mobilized from muscles and transferred to visceral organs and the tumor,^6^^,^^52^^,^^53^^,^^54^ suggesting that amino acid requirements may be increased as early as during pre-Cx tumor progression. In the growing spleen, we observed that signaling pathways regulating utilization and availability of amino acids were activated as early as the pre-Cx stage, probably to cope with tumor-induced immune cell proliferation. Consistently, previous studies in rats have demonstrated increased protein synthesis in the spleen during acute inflammation.^100^ However, although the activation of immune cell proliferation has an impact on amino acid requirements in inflammatory states, this demand has been estimated to be much lower than that associated with positive APP production by the liver.^101^ Liver amino acid uptake and protein synthesis have been shown to be increased in cancer patients and in tumor-bearing rodent models during cachexia.^54^^,^^102^^,^^103^^,^^104^^,^^105^ Given that, under normal conditions, the liver releases many amino acids that are consumed by the rest of the body, thereby regulating systemic amino acid availability,^74^^,^^75^ it is likely that the strong enhancement of amino acid utilization by the liver itself, due to increased positive-APP synthesis and/or gluconeogenesis, may affect circulating amino acid levels as early as the pre-Cx stage. Over time, the maintenance of a state of protein/amino acid hypermetabolism must raise a challenge in terms of amino acid supply to meet needs. During cachexia, amino acid release from muscle proteolysis may be expected to restore circulating concentrations of some amino acids to normal/basal levels or even higher, as previously observed for phenylalanine in Cx C26 mice.^97^^,^^106^ Consistently, we found that phenylalanine was the only amino acid with higher circulating levels at early cachexia compared to the pre-Cx state.

We found that pre-Cx reductions in systemic levels of amino acids, particularly all essential amino acids whose main fate is protein synthesis,^107^ were associated with a strong induction of positive-APP production, suggesting increased amino acid utilization by the liver as the main source of these exported proteins. However, unaltered rpS6 phosphorylation in livers of pre-Cx mice suggested that mTORC1-S6K1 axis-related protein synthesis regulation was unaffected at this stage. Interestingly, hepatic expression and production of negative APPs, including albumin, were shown to be reduced in tumor-bearing or infected rodents.^51^^,^^97^^,^^108^^,^^109^^,^^110^ While previous work has shown that *Alb* mRNA levels are reduced in Cx C26 mice,^97^ in the present study we found that they were already significantly reduced at the pre-Cx stage. Mechanistically, the TNF-α-CCAAT enhancer binding protein beta (C/EBPβ) axis has been shown to be involved in reducing albumin gene transcription.^110^^,^^111^^,^^112^ Given that albumin, the most abundant protein in plasma (around 35–40 mg/mL), accounts for 15% of hepatic protein synthesis^113^ and has an expression that is mainly regulated at the transcriptional level,^112^ this decrease in *Alb* mRNA levels probably allows a substantial saving of amino acids in the liver. Alternatively, increased hepatic autophagy could also have favored amino acid availability.

Persistent APR with continuous APP-positive production is likely to contribute to the etiology of cachexia.^114^^,^^115^^,^^116^ It is therefore important to characterize the kinetic evolution of hepatic protein/amino acid metabolism regulation during tumor progression before and upon induction of the syndrome. Data from the present study demonstrated activation of eIF2α signaling, a major regulator of protein/amino acid metabolism, in the liver at the onset of cachexia. In response to various cellular stresses, eIF2α phosphorylation results in both rapid inhibition of translation and increased expression of ATF4 target genes.^24^^,^^25^^,^^26^^,^^30^ At the whole organism level, this signaling pathway has been shown to play an important role in liver’s adaptive response to dietary restriction of essential amino acids.^63^^,^^117^^,^^118^ Our previous work has shown that the eIF2α-ATF4 signaling pathway is not activated by 24 h fasting.^119^ In the present study, the activation of eIF2α signaling may have resulted from sensing reduced or imbalanced amino acid availability or endoplasmic reticulum (ER) or mitochondrial stress associated with disrupted liver tissue homeostasis, through one or more eIF2α kinases. Consistently, increased expression of unfolded protein response (UPR) markers has been previously reported in the liver of Cx C26 mice,^120^ reinforcing the idea that adaptive mechanisms to cellular stress are triggered in this organ. These mechanisms may include adaptive upregulation of autophagy, to provide amino acids and/or remove damaged cellular components.^121^^,^^122^ In this study, we found that autophagy was upregulated in the livers of C26 mice as early as the pre-Cx stage, an effect that was enhanced at the onset of cachexia, with upregulation of ATF4-target autophagy genes. These findings support those of a previous study showing increased autophagy markers in the livers of Cx C26 mice.^120^ Given that eIF2α phosphorylation has been shown to promote autophagy, both through non-transcriptional effects and through ATF4-dependent activation of autophagy gene expression,^29^^,^^37^^,^^122^ data from the present study suggest a contribution of the eIF2α-ATF4 signaling pathway to increased hepatic autophagy from the onset of cachexia. Furthermore, the phosphorylation of eIF2α is known as an effective inhibitor of translation; however, we observed a concomitant marked increase in rpS6 phosphorylation in the liver at the onset of cachexia, consistently with previous studies having demonstrated an overall increase in hepatic protein synthesis in Cx rodents.^54^^,^^102^^,^^103^^,^^104^^,^^105^^,^^123^ On the other hand, cachexia was shown to be associated with a specific reduction in structural protein synthesis in the liver of colon cancer patients, concomitant with elevated hepatic production of positive APPs,^124^ suggesting the implementation of differential regulations of protein synthesis. Interestingly, recent data showed that translation attenuation following eIF2α phosphorylation is not uniform across all coding transcripts, preferentially affecting mRNAs encoding long-lived proteins.^125^ Such mechanisms could be involved in differential regulation of translation upon stress, depending on the function of the proteins synthesized. Following eIF2α phosphorylation, upregulated ATF4 activates the expression of specific target genes involved in adaptation to stress, including many genes involved in protein/amino acid metabolism.^24^^,^^25^^,^^26^^,^^30^ In our study, we show that expression of ATF4-regulated genes involved in amino acid transport, amino acid synthesis and autophagy was clearly increased at the onset of cachexia, suggesting a role of ATF4 in promoting amino acid availability in the liver. Interestingly, previous works have highlighted the possibility of a coordinated action between mTOR, as a kinase stimulating protein synthesis, and ATF4, as a transcription factor promoting amino acid availability, in anabolic settings in cultured mouse fibroblasts and *in vivo* in mouse liver.^126^^,^^127^ One can speculate that the implementation of such coordination could contribute to maintaining a high level of positive-APP production in the liver over the course of cachexia. Further studies are needed to functionally explore and precisely determine the role of the eIF2α-ATF4 signaling pathway in the liver during cancer progression, upon induction and evolution of cachexia.

It is known that the pathophysiology of cancer cachexia involves imbalances in protein and energy metabolism, driven by inflammation. However, better characterization and precise kinetics of early metabolic changes associated with tumor growth and cachexia induction are needed. Data from the present study highlight early cancer-related alterations in protein/amino acid homeostasis and activation of mechanisms regulating protein/amino acid metabolism in spleen and liver during progression to cachexia, likely involving the eIF2α-ATF4 signaling pathway. Exploring the role of eIF2α signaling in the regulation of whole-body protein/amino acid homeostasis during cancer progression will require further investigations.

### Limitations of the study

This study did not include measurements of overall hepatic protein synthesis. However, the data suggest that eIF2α phosphorylation-related attenuation of translation and mTOR/S6K1-dependent increase in protein synthesis may occur simultaneously in the liver at the onset of cachexia. This could indicate the implementation of differential regulations of synthesis of liver proteins according to their function. Thus, in parallel with measurements of overall protein synthesis, future studies should include characterization of such mechanisms and determining their role in regulating hepatic protein/amino acid metabolism during cancer progression, upon induction and evolution of cachexia.

Our data suggest an important role of the eIF2α-ATF4 signaling pathway as an adaptive regulatory mechanism of protein/amino acid metabolism in spleen and liver during the progression to cachexia. Functional studies will be required to assess the extent of its contribution, and should include analysis of amino acid import/export fluxes at the hepatic level.

As our study was carried out on male mice, the results cannot be generalized to females. Furthermore, we chose a mouse model of fairly acute cancer cachexia, with anorexia and body weight loss appearing rapidly after C26 cell implantation. Alterations in protein/amino acid homeostasis will need to be studied in other models, with detailed kinetic profile of parameters related to protein/amino acid metabolism before and at the onset of cachexia.

## Resource availability

### Lead contact

Further information and requests for resources and reagents should be directed to and will be fulfilled by the lead contact, Anne-Catherine Maurin (anne-catherine.maurin@inrae.fr).

### Materials availability

This study did not generate new unique reagents.

### Data and code availability

• Data supporting the findings of this study are included within the article or its supplemental information.
• This article does not report original code.
• Any additional information required to reanalyze the data reported in this paper is available from the lead contact upon request.

## Acknowledgments

This work was supported by the 10.13039/501100002915Fondation pour la Recherche Médicale (FRM-labeled team, DEQ20180339180, France) and the Institut National de Recherche pour l’Agriculture, l’Alimentation et l’Environnement (10.13039/501100022077INRAE, France). G.C. received financial support from the “10.13039/501100010115Région Auvergne-Rhône-Alpes” (CPER EPICURE, Nex-N-Mob, France); we thank Dr. Didier Rémond (Unité de Nutrition Humaine, INRAE, Université Clermont Auvergne, France) for his help in this respect. We thank the staff of the Digital PCR Platform of the CHU of Clermont-Ferrand (France), our research unit’s animal facility and the Mass Spectrometry Platform Facility of the Chemistry Institute of Clermont-Ferrand (France).

## Author contributions

Conceptualization, G.C., P.F., and A.-C.M.; formal analysis, G.C., L.P., M.L., C.B., I.P., L.M., P.F., L.B.B., and A.-C.M.; funding acquisition, P.F., D.T., and A.-C.M.; investigation, G.C., L.P., M.L., C.B., Y.D., and M.D.-M.; methodology, L.B.B., C.V., G.C., D.T., and A.-C.M.; project administration, A.-C.M.; resources, L.B.B.; supervision, J.H., P.F., and A.-C.M.; visualization, L.B.B., I.P., L.M., and P.F.; writing – original draft, G.C., P.F., M.L., C.B., and A.-C.M.; writing – review & editing, L.B.B., C.J., I.P., L.M., D.T., A.B., J.A., and L.C. All authors approved the version to be published.

## Declaration of interests

The authors declare no competing interests.

## STAR★Methods

### Key resources table

**Table:** 

REAGENT or RESOURCE	SOURCE	IDENTIFIER
**Antibodies**		

Rabbit monoclonal anti-phospho-(S51)-eIF2α	Abcam	Cat#ab32157; RRID: AB_732117
Rabbit polyclonal anti-eIF2α	Cell Signaling Technology	Cat#9722; RRID: AB_2230924
Rabbit polyclonal anti-phospho-(S240/244)-rpS6	Cell Signaling Technology	Cat#2215; RRID: AB_331682
Rabbit monoclonal anti-rpS6	Cell Signaling Technology	Cat#2217; RRID: AB_331355
Goat polyclonal anti-phospho-[Y705]-STAT3	Santa Cruz Biotechnology	Cat#sc-7993; RRID: AB_656682
Rabbit monoclonal anti-STAT3	Cell Signaling Technology	Cat#4904; RRID: AB_33126
Rabbit polyclonal anti-phospho-[S757]-ULK1	Cell Signaling Technology	Cat#6888; RRID: AB_10829226
Rabbit polyclonal anti-ULK1	Sigma-Aldrich	Cat#A7481; RRID: AB_1840703
Rabbit polyclonal anti-LC3B	Novus Biotechnology	Cat#NB100-2220; RRID: AB_10003146
Goat anti-rabbit	Cell Signaling Technology	Cat#7074; RRID: AB_2099233
Horse anti-mouse	Cell Signaling Technology	Cat#7076; RRID: AB_330924

**Chemicals, peptides, and recombinant proteins**		

Amino acid mixture-^13^ C,^15^N	Sigma-Aldrich	Cat#487910
TGX Stain-Free™ FastCast™ Acrylamide Kit, 10%	Bio-Rad Laboratories	Cat#1610183

**Critical commercial assays**		

ELISA Kit for mouse IL-6	R&D Systems	Cat#M6000B; RRID: AB_2877063
ELISA Kit for mouse GDF15	R&D Systems	Cat#DY6385; RRID: AB_3083003
ELISA Kit for mouse SAA	Tridelta Development Ltd	Cat#TP 802M
AccQ-Tag Ultra Derivatization Kit	Waters	Cat#186003836
Nucleospin RNA Kit	Macherey Nagel	Cat#740955
ddPCR Evagreen SuperMix	Bio-Rad Laboratories	Cat#186-4035
SSO Advanced Universal SYBR Green Supermix	Bio-Rad Laboratories	Cat#1725274

**Experimental models: Cell lines**		

C26 murine colon adenocarcinoma cells	Pr. Laure Bindels laboratory	N/A

**Experimental models: Organisms/strains**		

Mouse: CD2F1 male mice	Charles River Laboratories	Cat#033CDF-1; RRID:IMSR_CRL:033

**Oligonucleotides**		

See Table S1 for qPCR primers	Sigma-Aldrich Genosys	N/A

**Software and algorithms**		

ImageJ v1.54	ImageJ	https://imagej.net/ij/; RRID:SCR_003070
Prism 9.0	GraphPad	https://www.graphpad.com/; RRID:SCR_002798
QX Manager, Standard Edition, v 1.2.345	Bio-Rad Laboratories	https://www.bio-rad.com/fr-fr/life-science/digital-pcr/qx-software

### Experimental model and study participant details

All mandatory laboratory health and safety procedures have been complied with in the course of conducting any experimental work reported in this paper.

#### Cell culture

C26 is a murine colon adenocarcinoma that was initially induced by N-nitroso-N-methylure, known to induce cachexia in syngeneic hosts.^38^ C26 cells (provided by Pr. L. B. Bindels) were maintained in high-glucose Dulbecco’s modified Eagle’s medium, supplemented with 10% fetal bovine serum and 100 units/ml penicillin and streptomycin, at 37°C with 5% CO2.

#### Animal experiments

All animal experiments were carried out in accordance with the guidelines of the local ethics committee (CEEA-02), as well as French and European Union laws: permission to experiment on mice APAFIS#24596–2020012713544797 v5 and animal facilities agreement E6334515. To describe animal experiments, we have taken into account the recommendations of the ARRIVE Guidelines for Reporting Animal Research.^128^ We used the C26 mouse model of cancer cachexia, which is associated with both anorexia, body weight reduction and loss of muscle mass.^19^^,^^55^ Eight-week-old CD2F1 male mice were purchased from Charles River Laboratories, France. They were housed individually in the presence of an environmental enrichment element (a plastic tube-shaped cache), at an ambient temperature of 22°C and according to a standard 12h/12h light/dark cycle. Throughout the experiment, mice had free access to standard food (Safe Cat# A03, 52% carbohydrate, 5.1% lipids, and 21.4% proteins) and tap water. After sixteen days of acclimatization, animals were assigned to the different groups so as to obtain a homogeneous distribution of animals according to body weight. The average body weight was 24.9 g at the time of intervention. Mice were inoculated subcutaneously and intra-scapularly with 10^6^ C26 cells in 0.15 mL of NaCl 9/1000 (*n* = 13). Control mice were injected with 0.15 mL of NaCl 9/1000 (sham-injected mice; *n* = 9). These procedures were performed in the same session for all mice. Food intake and body weight were measured every 24 h (this measurement was always performed at the same time at the end of the dark period). Food consumption was monitored using specific cages developed by our team, equipped with removable external feeders including crumb collectors, allowing precise measurement of food intake. Time points of sacrifice by necropsy were determined accordingly to our objective to analyze and compare two stages: before the onset of cachexia (pre-Cx) on day 6 post-implantation of C26 cells and at the onset of cachexia (Cx) on day 8. The sham-injected group combined control mice on days 6, 7 and 8 post-injection of NaCl (we observed no significant variation between samples taken on different days for any of the parameters analyzed in the sham-injected group). Sacrifices were performed 5–6 h after the beginning of the light period. Blood was collected by cardiac puncture under deep general anesthesia of the animals with isoflurane, then treated with EDTA. Plasma samples were obtained by centrifugation and stored at −80°C until use. The animals were then subjected to death by cervical dislocation before tumors, tissues and organs were harvested, weighed and frozen on dry ice and stored at −80°C until processing.

### Method details

#### RNA extraction & cDNA synthesis

Tissues were powdered on dry ice (except hypothalamus), then crushed using a vibrating shredder (Retsch MM 400) in the first extraction step. Total RNA was isolated using Nucleospin RNA Kit from Macherey Nagel (Cat#740955) and treated with DNase I, Amp Grade (Invitrogen, Cat#18068015) prior to cDNA synthesis. After RNA extraction, RNA concentration was measured using a NanoDrop One (ThermoFisher Scientific). RNA (0.5 μg) was reverse transcribed with 100 U of Superscript II Reverse Transcriptase (Invitrogen, Cat#18064014) using 100 μM random hexamer primers (Invitrogen, Cat#48190011), according to the manufacturer’s instructions.

#### Digital qPCR

Droplet digital PCR (ddPCR) was performed using a QX200 Droplet Digital PCR system (Bio-Rad Laboratories). The ddPCR reactions were carried out into a final volume of 20 μL comprising 10 μL of 2X QX200 ddPCR Evagreen SuperMix (Bio-Rad Laboratories, Cat#186–4035), 0.1 μM each of forward and reverse primers, and 5 μL cDNA. For primer sequences, see Table S1. Droplets were generated in eight-well cartridges using the QX200 droplet generator (Bio-Rad Laboratories) prior to PCR amplification on the C1000 Thermal Cycler (Bio-Rad Laboratories). The PCR parameters were as follows: initial denaturation/activation step at 95°C for 10 min then 50 cycles of 30 s denaturation at 95°C, and annealing/extension at 57°C for 1 min followed by 98°C for 10 min and a final hold at 4°C. After PCR the droplets were individually analyzed using the QX200 droplet reader (Bio-Rad Laboratories) and data were processed with QX Manager Software, Standard Edition, v 1.2.345. Only reactions generating more than 10,000 droplets were considered valid.

#### Conventional qPCR

Real-time quantitative PCR was performed on a Bio-Rad CFX-96 detection system with quantitative PCR SSO Advanced Universal SYBR Green Supermix (Bio-Rad Laboratories, Cat#1725274) and with a primer concentration of 0.5 μM. PCR conditions were standardized to 40 cycles of 95°C for 10 s and 59°C for 30 s with the primers (Sigma-Aldrich Genosys) for specific mouse mRNA sequences. For primer sequences, see Table S1. Quantification of mRNA expression was performed according to the delta-delta Ct method.

#### Protein extraction

Tissues were powdered on dry ice, then crushed using a vibrating shredder (Retsch MM 400) in the first extraction step. Total proteins were extracted from liver samples with 1X Laemmli buffer, as previously described.^37^ Protein extracts were prepared from spleen tissue using RIPA buffer as previously described.^127^ Protein samples were boiled at 95°C for 10 min before electrophoresis.

#### Western-blot analysis

Proteins were resolved by SDS-PAGE on 10% TGX-polyacrylamide gels (TGX Stain-FreeFastCast Acrylamide Kit, Bio-Rad Laboratories Cat#1610183) in Tris-glycine-SDS buffer. Protein transfer was performed at 4°C in Tris-glycine buffer onto polyvinylidene fluoride (PVDF) membranes (Amersham Hybond P0.45, Cat# 10600023). We used the TGX system for non-specific detection of all proteins. This signal was acquired both on electrophoresis gels after migration (not shown) in order to check the quantity of loaded proteins, but also on PVDF membranes just after transfer for controlling its efficiency. Membranes were blocked with non-fat dried milk powder (5% w/v) diluted in TBS-Tween for 1 h, then incubated with primary antibodies diluted in TBS-Tween containing 5% BSA (dilution according to manufacturer’s instructions) overnight at 4°C, then washed 3 times for 5 min in TBS-Tween. HRP-coupled anti-species secondary antibodies were diluted at 1:2000 in TBS-Tween containing 5% (w/v) non-fat dried milk and used at room temperature for 1 h. Membranes were washed 3 times for 5 min in TBS-Tween, then rinsed in TBS before analysis. Luminata western HRP substrate (Millipore, WBLUR0500) and a chemi-luminescence imager (G:box, Syngene) were used to detect signals. Signal intensities were quantified using the ImageJ software (RRID: SCR_003070). For a given sample, the respective intensities of LC3-I and LC3-II signals were quantified from the same selected area. Relative quantifications have been performed, with mean control condition value normalized to 1.

#### Antibodies

Anti-phospho-(S51)-eIF2α antibody was from Abcam (Cat#ab32157; RRID: AB_732117) and anti-phospho-[Y705]-STAT3 antibody from Santa Cruz Biotechnology (Cat#sc-7993; RRID: AB_656682). Antibodies targeting eIF2α (Cat#9722; RRID: AB_2230924), phospho-[S757]-ULK1 (Cat#6888; RRID: AB_10829226), phospho-(S240/244)-rpS6 (Cat#2215; RRID: AB_331682), rpS6 (Cat#2217; RRID: AB_331355) and STAT3 (Cat#4904; RRID: AB_331269) were from Cell Signaling Technology, as well as anti-species secondary antibodies coupled to horseradish peroxidase: anti-rabbit antibody (Cat#7074; RRID: AB_2099233) and anti-mouse antibody (Cat#7076; RRID: AB_330924). Anti-LC3B antibody was purchased from Novus Biotechnology (Cat#NB100-2220; RRID: AB_10003146) and anti-ULK1 from Sigma-Aldrich (Cat#A7481; RRID: AB_1840703).

#### ELISA

IL-6, GDF15 and SAA were detected in EDTA-treated plasma samples using commercially available ELISA kits (R&D Systems, Cat#M6000B, RRID: AB_2877063 for mouse IL-6; R&D Systems, Cat#DY6385, RRID: AB_3083003 for mouse GDF15; Tridelta Development Ltd, Cat#TP 802M, for mouse SAA), according to the manufacturer’s instructions.

#### Plasma amino acid analysis

For sample preparation, 25 μL of plasma were vortexed with 25 μL of the internal standard solution (mixture of labeled AA isotopes; Sigma-Aldrich, Cat#487910) and 150 μL of methanol with 0.1% formic acid at −20°C. The extract was left at −20°C during 30 min to precipitate the proteins then centrifuged at 14000 rpm for 10 min. After that, for derivatization reaction, 10 μL of the supernatant was mixed with 70 μL of borate buffer (pH 8.8) and 20 μL of the AQC solution (AccQ·Tag Ultra Derivatization Kit, Waters, Cat#186003836) and the reaction mixtures were heated at 55°C for 10 min before LC/MS analysis. A standard mixture solution contained 100 mg/L of each amino acid was prepared in water then calibration solutions were realized by dilution in water at concentrations between 0.1 and 100 mg/L. Before LC/MS analysis, 25 μL of each calibration solution was taken to derivatization reaction with the same method as plasma sample. Chromatographic separation was performed with an Ultimate 3000 UHPLC (Thermo Scientific) and a CORTECS C18 (150 mm × 2.1 mm; 1.6μm) column (Phenomenex) maintained at 55°C. The mobile phases consisted of water (A) and acetonitrile (B), both with 0.1% formic acid. The LC gradient used was as follows: from 99% A hold for 1 min, then to 87% A in 1 min, then to 85% A in 3.5 min; then to 5% A in 1 min finally 5% A was maintained 1 min and went back to the initial conditions within 0.1 min for an equilibration time of 1.4 min. The flow rate was 0.5 mL/min and the injection volume was 5 μL. The detection was performed with a Q Exactive mass spectrometer (Thermo Scientific) using positive electrospray ionizations, in SRM mode. The desolvation temperature was 438°C and capillary temperature was 320°C. The gas (N_2_) flow rates (arbitrary unit) were set at 53 for Sheath gas, 14 for Aux gas and 3 for Sweep gas. The spray voltage was 3.5 kV with an S Lens level RF Level at 60. The mass resolution was set at 35000, automatic gain control at 10^6^ and injection time at 50 ms.

### Quantification and statistical analysis

Groups were composed of *N* = 9 NaCl-injected mice for the Sham group, *N* = 7 C26-injected mice for the Pre-Cx group and *N* = 6 C26-injected mice for the Pre-Cx group. No mice were excluded from the study. Data are presented as mean ± standard error of the mean (SEM). To evaluate the significance of the difference of both food intake and body weight between groups over time, two-way analyses of variance (Anova) were performed for each of these parameters and completed by Tukey’s multiple comparison tests to analyze differences between groups at each time point (see the legend of Figure 1). For other analyses, one-way Anova followed by Tukey’s multiple comparisons were performed to compare and assess the significance of differences between the three groups with each other. Statistical analyses were performed using GraphPad Prism 9.0 (RRID:SCR_002798). *p* < 0.05 was considered statistically significant (∗*p* < 0.05; ∗∗*p* < 0.01; ∗∗∗*p* < 0.001; ∗∗∗∗*p* < 0.0001). In some cases, *t-*tests were performed to assess the significance of differences by pairwise comparison between two specific groups (mentioned in the text; #*p* < 0.05 by t-test).
