# Supplementary material for: Pre-cachectic changes in amino acid homeostasis precede activation of eIF2α signaling in the liver at the onset of C26 cancer-induced cachexia
Source: iScience. 2025 Feb 14;28(3):112030. doi: 10.1016/j.isci.2025.112030 (PMC11928868; doi:10.1016/j.isci.2025.112030)
Supplement: Document S1. Figures S1–S3 and Table S1 [file mmc1.pdf]

iScience, Volume *28*

Supplemental information

Pre-cachectic changes in amino acid homeostasis precede activation of eIF2α signaling in the liver at the onset of C26 cancer-induced cachexia

Ghita Chaouki, Laurent Parry, Cyrielle Vituret, Céline Jousse, Martin Leremboure, Céline Bourgne, Laurent Mosoni, Yoann Delorme, Mehdi Djelloul-Mazouz, Julien Hermet, Julien Averous, Alain Bruhat, Lydie Combaret, Daniel Taillandier, Isabelle Papet, Laure B. Bindels, Pierre Fafournoux, and Anne-Catherine Maurin

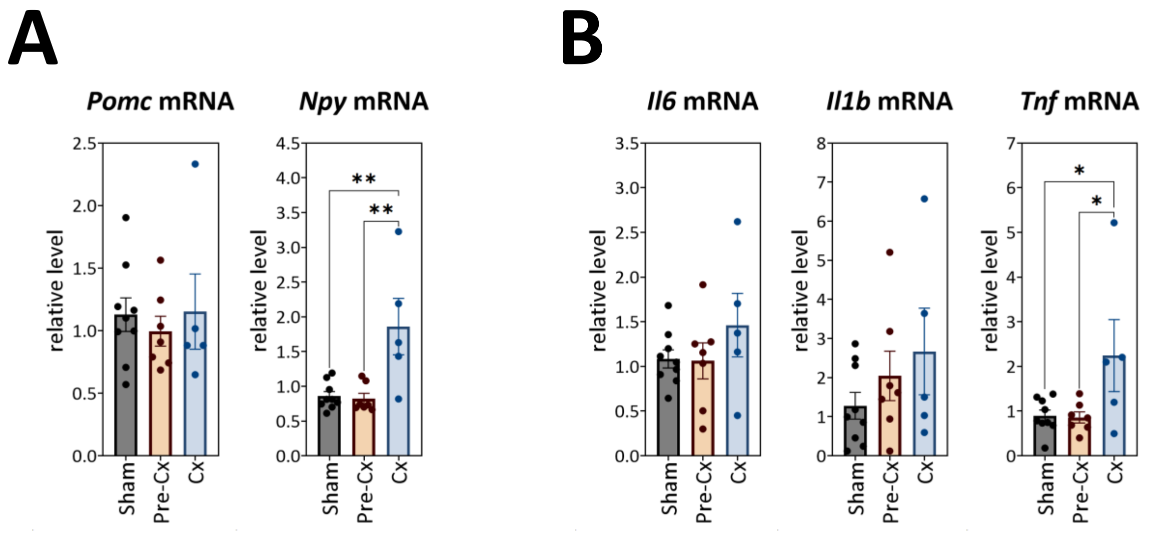

Figure S1: Onset of cachexia was associated with increased expression of *Tnf* and *Npy* in the hypothalamus (Related to Figure 1). Relative mRNA levels of *Pomc*, *Npy*, *Il6*, *Il1b* and *Tnf* were measured in the whole hypothalamus. mRNA levels were expressed relative to *Ppia* mRNA level. The significance of differences between groups was assessed by one-way analysis of variance followed by Tukey's multiple comparisons (* $P < 0.05$; ** $P < 0.01$).

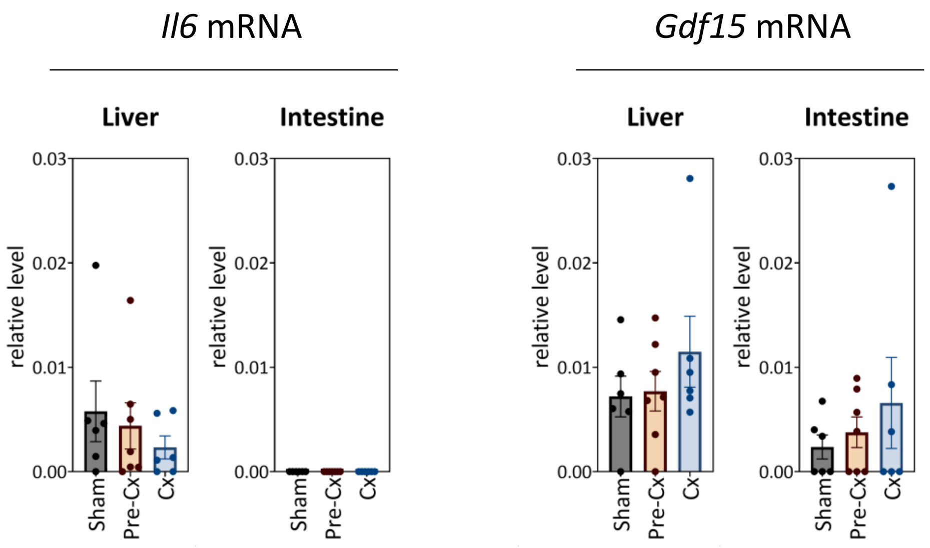

Figure S2: Relative mRNA levels of *Il6* and *Gdf15* in the liver and intestine (Related to Figure 2). mRNA levels of *Il6* and *Gdf15*, as determined by digital qPCR, were expressed relative to *Ppia* mRNA level. The significance of differences between groups was assessed by one-way analysis of variance followed by Tukey's multiple comparisons.

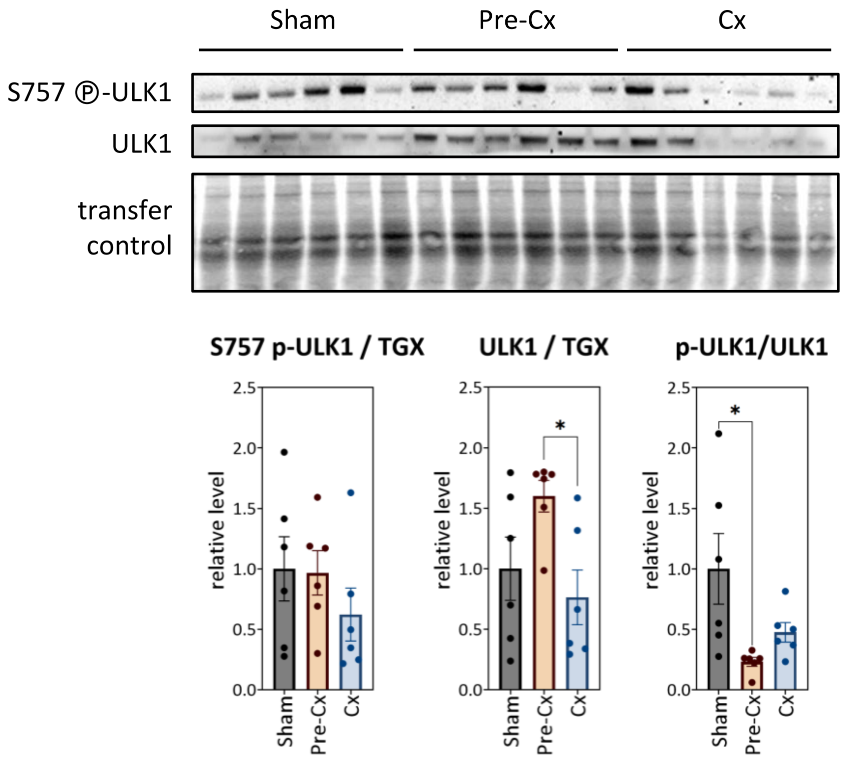

Figure S3: Analysis of [Ser757]-ULK1 phosphorylation in the liver (Related to Figure 6). Expression levels of ULK1 and phospho-[Ser757]-ULK1 were analyzed by western-blot. TGX™ signals were used to control protein transfer. The significance of differences between groups was assessed by one-way analysis of variance followed by Tukey's multiple comparisons (* $P < 0.05$).

Gene	Gene ID	Sequence
Ppia	268373	***F:*** TCGAGCTCTGAGCACTGGA
		R: CAGTGCCATTATGGCGTGTA
Il6	16193	***F:*** TCCTAACAGATAAGCTGGAGT
		R: GCTTAGGCATAACGCACTAG
Tnfa	21926	***F:*** CGACTACGTGCTCCTCACC
		R: CTCCAGCTGGAAGACTCCT
Il1b	16176	***F:*** GCTCTCCACCTCAATGGAC
		R: GATCCACACTCTCCAGCTG
Gdf15	23886	***F:*** TCCTGCTGTTCCTGCTGCT
		R: GGTCGCTGTTCAGGCATTG
Saa1	20208	***F :*** CATTTGTTCACGAGGCTTTCC
		R : GTTTTTCCAGTTAGCTTCCTTCATGT
Saa2	20209	***F :*** GGGGTCTGGGCTTCCTATCT
		R : CCATTCTGAAACCCTTGTGG
Apcs	20219	***F :*** AGCCCCACCCAGTATAGTCC
		R : GGTCAGCACATAGTCCCACA
Map1lc3b	67443	***F:*** GAGCGAGTTGGTCAAGATCA
		R: GGAGGCGTAGACCATGTAG
Atg12	67526	***F:*** TAAACTGGTGGCCTCGGAAC
		R: CCATCACTGCCAAAACACTCA
Atg16l1	77040	***F:*** AGATGAATGAAGCAAAGATT
		R: AGTAAAAGTAATCTGCAGGG
Sqstm1	18412	***F:*** TGGGCAAGGAGGAGGCGACC
		R: CCTCATCGCGGTAGTGCGCC
Slc38a2	67760	***F:*** CGTTCGCCTTTGTGTATCAA
		R: GGAGGGTAGGGTACTGCACA
Slc7a5	20539	***F:*** CCTCTTCCTCATTGCCGTGT
		R: GGGCTTGTTCTTCCACCAGA
Slc1a5	20514	***F:*** GTACCACATAATCCAGGAG
		R: GTGGTCTTCGCTATCGTC
Slc7a11	26570	***F:*** GCATGTCCCTGGTTTTCTGG
		R: AAGCCAGCAAAGGACCAAAG
Ddit3	13198	***F:*** CCTAGCTTGGCTGACAGAGG
		R: CTGCTCCTTCTCCTTCATGC
Asns	27053	***F:*** TACAACCACAAGGCGCTACA
		R: AAGGGCCTGACTCCATAGGT
Trib3	228775	***F:*** CCAGAGATACTCAGCTCCCG
		R: GAGGAGACAGCGGATCAGAC
Atf3	11910	***F:*** CCAGGTCTCTGCCTCAGAAG
		R: CATCTCCAGGGGTCTGTTGT
Pomc	18976	***F:*** CATTAGGCTTGGAGCAGGTC
		R: GTTCTTGAAGAGCGTCACCA
Npy	109648	***F:*** AGAGGACATGGCCAGATACT
		R: GTCTTCAAGCCTTGTTCTGG
Trim63	433766	***F:*** ACGAGAAGAAGAGCGAGC
		R: CTTGGCACTTGAGAGGAA
Fbxo32	67731	***F:*** ATGCACACTGGTGCAGAGAG
		R: TGTAAGCACACAGGCAGGTC
Ctsl	13039	***F:*** GCAGCAAGAACCTCGACCAT
		R: GTTGTCCCGGTCTTTGGCTA
Ywhaz	22631	***F:*** CTGGCCCTCAACTTCTCTGT
		R: AATGGCTTCATCGAAAGCTG
Actb	11461	***F:*** TACAGCTTCACCACCACAGC
		R: AAGGAAGGCTGGAAAAGAGC

Table S1: Sequences of primers used for qPCR.
